# Hospital care patterns among patients with psychotic-disorder coding and metastatic/secondary malignant neoplasm coding: A nationwide administrative cohort study in Spain

**DOI:** 10.1007/s00520-026-11440-0

**Published:** 2026-11-04

**Authors:** Belén Sánchez-Barriguete, Carlos Navarro-García

**Affiliations:** 1 Hospital San Juan de Dios, Ciempozuelos, Madrid, Spain; 2 Facultad de Ciencias Biomédicas y de la Salud, Universidad Alfonso X El Sabio (UAX), Madrid, Spain; 3 Programa de Doctorado en Ciencias de la Salud, Escuela Internacional de Doctorado, Universidad Rey Juan Carlos, Madrid, Spain

**Keywords:** Psychotic disorder, Schizophrenia spectrum disorders, Metastatic cancer, Palliative care, Hospital care, Chemotherapy coding, Administrative data

## Abstract

**Purpose:**

Patients with psychotic disorders may experience differences in cancer care, but evidence on hospital supportive and palliative care patterns in advanced cancer remains limited. We examined whether psychotic-disorder coding was associated with hospital palliative-care coding and selected hospital treatment-intensity markers among patients with metastatic/secondary malignant neoplasm coding in a Spanish hospital oncology extract.

**Methods:**

We conducted a nationwide retrospective cohort study using Spanish hospital administrative data from 2021 to 2024. The source extract was a requested oncology administrative hospital extract, composed overwhelmingly of episodes with malignant cancer recorded as the principal diagnosis and also containing a small subset of palliative-care principal-diagnosis episodes with malignant neoplasm/metastatic coding in secondary fields. Eligible patients had secondary malignant neoplasm/metastatic disease coding in secondary diagnosis fields (C77-C79). The first metastatic-coded episode observed during 2021–2024 was treated as an administrative index episode and not necessarily as the onset of metastatic disease. The unit of analysis was the patient. For each patient, a metastatic care window was defined from the first metastatic-coded hospital episode to the last available episode. Psychotic-disorder coding was defined primarily as F20-F29 within this window; a temporal audit examined F20-F29 coding before or at the index episode. Outcomes were considered present if coded at least once during the metastatic window. Multivariable logistic regression adjusted for age, sex, year, tumor group, autonomous community, hospital service, and approximate non-oncologic Charlson-based comorbidity measured at the first metastatic-coded episode.

**Results:**

The cohort included 272,809 patients, of whom 1989 had psychotic-disorder coding within the metastatic care window (0.73%). A temporal audit identified 1938 patients with F20-F29 coding before or at the first metastatic-coded episode in the observed extract; 1889 patients met both definitions, 100 had F20-F29 coding in the metastatic care window only after the index episode, and 49 had F20-F29 coding before or at the index episode but not within the metastatic care window. Psychotic-disorder coding was associated with higher odds of hospital palliative-care coding (OR 1.35, 95% CI 1.19–1.52) and mechanical ventilation (OR 1.44, 95% CI 1.15–1.82). In-hospital chemotherapy procedure coding showed lower adjusted odds among patients with psychotic-disorder coding (OR 0.73, 95% CI 0.59–0.91). Marginal adjusted probabilities for palliative-care coding were 20.6% with psychotic-disorder coding versus 17.0% without psychotic-disorder coding. Corresponding probabilities were 4.5% versus 5.8% for in-hospital chemotherapy procedure coding and 3.6% versus 2.6% for mechanical ventilation.

**Conclusion:**

In this nationwide hospital administrative oncology extract, psychotic-disorder coding was associated with distinct hospital coding patterns after metastatic/secondary malignant neoplasm coding. The findings should be interpreted as hospital-coded associations and not as direct measures of appropriateness, treatment quality, outpatient systemic therapy, or specialist palliative-care access.

**Supplementary Information:**

The online version contains supplementary material available at https://doi.org/10.1007/s00520-026-11440-0.

## Introduction

People living with psychotic disorders have higher morbidity and mortality and may face structural barriers across the cancer care continuum [1, 2]. In oncology, these barriers can operate through delayed diagnosis, fragmented follow-up, differences in treatment decision-making, comorbidity burden, communication challenges, social vulnerability, and clinical uncertainty when psychiatric symptoms coexist with advanced disease [3–5].

Supportive care is central to the management of metastatic cancer. It includes symptom control, palliative care integration, management of treatment complications, and decisions about potentially intensive interventions. Metastatic disease is the clinical setting in which supportive and palliative care decisions are especially relevant and more consistently documented in hospital records. However, less is known about whether patients with psychotic disorders have different patterns of hospital-based supportive, palliative, and oncologic care once metastatic/secondary malignant neoplasm coding is present. Previous reviews have emphasized that palliative care for people with severe or persistent mental illness, including schizophrenia spectrum disorders, remains underdeveloped and inconsistently integrated across care systems [6–12].

This question is clinically relevant because differences in care patterns may reflect appropriate individualized decisions, differences in clinical status, barriers to access, or inequities in treatment pathways. Population-based studies have reported excess cancer mortality and treatment disparities among people with mental illness, including schizophrenia and other severe mental disorders [13–19]. Administrative hospital data cannot adjudicate appropriateness at the individual level, but they can identify population-level signals that require clinical and policy attention.

We therefore conducted a nationwide retrospective cohort study using a Spanish hospital oncology administrative extract to examine whether psychotic-disorder coding was associated with hospital palliative-care coding among patients with metastatic/secondary malignant neoplasm coding. As exploratory secondary objectives, we evaluated selected hospital treatment-intensity markers during the metastatic care window, including intensive care unit admission, in-hospital chemotherapy procedure coding, mechanical ventilation, transfusion, and tracheostomy.

## Methods

### Study design and data source

We performed a retrospective cohort study using Spanish hospital administrative data from 2021 to 2024, called RAE-CMBD^1^. The source database was a requested oncology administrative extract for the study period, composed overwhelmingly of episodes with malignant cancer recorded as the principal diagnosis and including a small subset of palliative-care principal-diagnosis episodes with malignant neoplasm/metastatic coding in secondary fields. Diagnoses and procedures were coded using ICD-10-CM/PCS-derived Spanish coding fields. Patients were linked using the recoded patient identifier supplied in the RAE-CMBD extract. We treated this official linkage variable as the longitudinal patient key across observed episodes; the extract did not include documentation allowing us to independently audit the identifier-generation algorithm.

The extract does not represent all people with metastatic cancer in Spain or all hospitalizations experienced by those patients. It contains hospital episodes included in the requested oncology extract, which was composed overwhelmingly of episodes where malignant cancer was recorded as the principal diagnosis. Admissions primarily coded for infection, respiratory failure, psychiatric deterioration, or other non-cancer principal diagnoses may therefore be absent.

No hospital identifier or death date was available in the analysis extract. The extract included autonomous community and discharge-status fields, but not a validated date of death.

The study is reported in accordance with the STROBE Statement [20].

### Study population

The unit of analysis was the patient. Patients were identified using the recoded patient identifier available in the database. We included patients with metastatic/secondary malignant neoplasm coding, defined as at least one hospital episode with a secondary diagnosis code C77, C78, or C79. Secondary diagnosis fields were used because the source extract was an oncology administrative extract in which the principal diagnosis generally captured the primary tumor or, for a small subset, palliative-care coding; secondary diagnosis fields captured secondary malignant neoplasm coding while preserving the principal-diagnosis field for tumor grouping.

Patients whose administrative index episode had Z51.5 as the principal diagnosis were retained only when C77-C79 metastatic/secondary malignant neoplasm coding was present in secondary diagnosis fields; these patients represented 4814 patients (1.8% of the analytic cohort).

Codes C77-C79 were used to define the primary cohort. Because C77 may include lymph-node secondary malignant neoplasm coding that does not always correspond to distant stage IV disease, we performed a sensitivity analysis restricted to C78-C79.

For each patient, we defined a metastatic care window starting at the first hospital episode with secondary malignant neoplasm/metastatic disease codes and ending at the last available hospital episode during the study period. The first metastatic-coded episode observed in 2021–2024 was an administrative index episode. Patients may therefore have had newly documented or prevalent metastatic disease.

The metastatic care window was used to align the exposure and outcomes with the period in which advanced cancer was documented in hospital records. We considered outcomes present if they were coded at least once during this window because the events of interest may occur in different admissions. Restricting the analysis to the first metastatic-coded episode would have underestimated patient-level exposure to these forms of care and would have treated subsequent hospital information as irrelevant despite the longitudinal nature of metastatic cancer care.

### Exposure

The primary exposure was psychotic-disorder coding, defined as any secondary diagnosis code F20-F29 within the metastatic care window. These codes include schizophrenia, schizotypal disorder, persistent delusional disorders, acute and transient psychotic disorders, schizoaffective disorders, and other non-organic psychotic disorders. The exposure was assigned at patient level to avoid treating multiple admissions from the same person as independent observations.

Because this definition does not by itself establish temporal precedence, we audited F20-F29 coding before or at the first metastatic-coded episode in the observed extract. We also described the distribution of individual F20-F29 diagnostic groups and specified sensitivity analyses focused on schizophrenia/schizoaffective disorder coding.

### Outcomes

The primary outcome was hospital palliative-care coding during the metastatic care window, defined by code Z51.5/Z515 in any diagnosis field. This outcome indicates hospital palliative-care coding and does not identify specialist palliative-care referral, timing, content, intensity, quality, or preference-concordance.

Secondary outcomes, all measured during the metastatic care window, were intensive care unit admission, in-hospital chemotherapy procedure coding, mechanical ventilation, transfusion, and tracheostomy. Intensive care unit admission was based on the administrative ICU field. Procedure-based outcomes were identified using prespecified ICD-10-PCS-derived procedure algorithms. In-hospital chemotherapy procedure coding does not capture outpatient chemotherapy, day-hospital treatment, immunotherapy, targeted therapy, oral anticancer therapy, or all systemic anticancer treatment. Detailed definitions are provided in the supplementary appendix.

### Covariates

Models adjusted for age, sex, calendar year, tumor group based on the primary diagnosis, autonomous community, hospital service, and approximate non-oncologic Charlson-based comorbidity score, all measured at the first metastatic-coded episode. These covariates were selected a priori because they capture demographic factors, cancer type, temporal trends, territorial and organizational differences in hospital care, and non-oncologic comorbidity burden. Oncologic Charlson components were not included in the comorbidity score because the cohort was defined by metastatic/secondary malignant neoplasm coding [21, 22].

### Statistical analysis

Patient characteristics and hospital outcomes were summarized overall and by psychotic-disorder coding status. Categorical variables are presented as counts and percentages, and continuous variables as mean and standard deviation.

We fitted multivariable logistic regression models for each outcome. The main parameter of interest was the adjusted odds ratio for psychotic-disorder coding. The primary analysis used the full patient cohort and outcomes defined within the metastatic care window. Categorical covariates were included using indicator variables. Patients with missing age were excluded from complete-case model fitting; missing or unavailable categorical values were retained as explicit missing/not-recorded categories where applicable.

We estimated marginal adjusted probabilities by setting psychotic-disorder coding to present and absent for all patients in the fitted model and averaging predicted probabilities. Adjusted risk differences were calculated as the difference between these marginal probabilities. Two-sided p values and 95% confidence intervals are reported. For secondary outcomes, interpretation prioritized effect size, direction, and confidence intervals rather than *p* values alone, and these outcomes were treated as exploratory.

We performed sensitivity analyses restricted to patients with C78-C79 coding, using psychotic-disorder coding before or at the index episode, and omitting hospital service. Hospital service at the first metastatic-coded episode was included in the main model to account for baseline hospital context, but it may partly represent the pathway under study; therefore, we added a sensitivity analysis omitting hospital service. Hospital-level cluster-robust standard errors could not be estimated because no hospital identifier was available. Models adjusted for autonomous community, but residual hospital-level clustering and coding-practice variation remain possible.

All reported logistic regression models converged. Sparse exploratory outcomes, particularly tracheostomy, were interpreted cautiously because low event counts can make estimates less stable.

## Results

### Cohort

The source dataset contained 1,224,317 hospital episodes in the requested oncology extract during the study period; 1,206,045 episodes (98.5%) had malignant cancer recorded as the principal diagnosis. After identifying patients with metastatic/secondary malignant neoplasm coding in secondary diagnosis fields, the analytic cohort included 272,809 patients (Table 1).

**Table 1:** Participant flow and cohort accounting

Step	Records/patients
Source hospital episodes in oncology extract	1,224,317 episodes
Patients with secondary malignant neoplasm/metastatic coding C77-C79	272,809 patients
Patients with psychotic-disorder coding F20-F29 in metastatic care window	1989 patients
Patients with F20-F29 before or at first metastatic-coded episode	1938 patients
Patients with both F20-F29 in the window and before/at first metastatic-coded episode	1889 patients
Patients with F20-F29 in the window only after first metastatic-coded episode	100 patients
Patients with F20-F29 before/at first metastatic-coded episode but not in the window	49 patients
Patients retained in C78-C79 restricted sensitivity cohort	205,136 patients

Psychotic-disorder coding was present in 1989 of 272,809 patients (0.73%). A temporal audit identified 1938 patients with F20-F29 coding before or at the first metastatic-coded episode in the observed extract. These temporal categories were not fully nested: 1889 patients had F20-F29 coding both within the metastatic care window and before or at the index episode, 100 had F20-F29 coding in the metastatic care window only after the index episode, and 49 had F20-F29 coding before or at the index episode but not within the metastatic care window. Among patients with F20-F29 coding in the metastatic care window, the diagnostic-group distribution was: F20 1,421; F21 14; F22 221; F23 18; F25 184; F28 2; F29 164; and no patients coded with F24, F26, or F27. Diagnostic groups were not mutually exclusive because individual patients could have codes from more than one F20-F29 category during the metastatic care window.

Patients with psychotic-disorder coding were younger on average and were more often male. Lung cancer was more frequent among patients with psychotic-disorder coding than among those without psychotic-disorder coding.

Observation opportunities were similar by psychotic-disorder coding status. Patients without psychotic-disorder coding had a mean of 1.43 eligible episodes (median 1, IQR 1–2) and mean observed window duration of 68.6 days (median 11, IQR 5–39). Patients with psychotic-disorder coding had a mean of 1.44 eligible episodes (median 1, IQR 1–2) and mean observed window duration of 62.6 days (median 15, IQR 6–41) (Table 2).

**Table 2:** Observation opportunity during the metastatic care window by psychotic-disorder coding status

Psychotic disorder	Episodes, mean (SD)	Episodes, median (IQR)	Observed days, mean (SD)	Observed days, median (IQR)
No	1.43 (1.19)	1 (1–2)	68.6 (160.5)	11 (5–39)
Yes	1.44 (0.80)	1 (1–2)	62.6 (148.2)	15 (6–41)

### Patient characteristics and hospital outcomes

Table 3 summarizes patient characteristics and hospital outcomes by psychotic-disorder coding status. Tumor groups are summarized in the main table and shown completely in the supplementary material so that categories sum to the analytic cohort total; not-recorded sex is explicitly displayed.

**Table 3:** Patient characteristics and hospital outcomes by psychotic-disorder coding status

Characteristic	Total	No psychotic disorder	Psychotic disorder
Patients, *n*	272,809	270,820	1989
Age, mean (SD)	67.3 (13.7)	67.4 (13.7)	62.9 (11.2)
Sex: Male	143,810 (52.7%)	142,672 (52.7%)	1138 (57.2%)
Sex: Female	128,997 (47.3%)	128,146 (47.3%)	851 (42.8%)
Sex: Not recorded	2 (0.0%)	2 (0.0%)	0 (0.0%)
Tumor group: Other cancer	67,987 (24.9%)	67,564 (24.9%)	423 (21.3%)
Tumor group: Lung	48,160 (17.7%)	47,590 (17.6%)	570 (28.7%)
Tumor group: Colorectal	40,250 (14.8%)	39,996 (14.8%)	254 (12.8%)
Tumor group: Breast	28,940 (10.6%)	28,736 (10.6%)	204 (10.3%)
Tumor group: Pancreas	17,399 (6.4%)	17,292 (6.4%)	107 (5.4%)
Tumor group: Stomach	11,530 (4.2%)	11,465 (4.2%)	65 (3.3%)
Other tumor groups	58,543 (21.5%)	58,177 (21.5%)	366 (18.4%)
Approximate non-oncologic Charlson-based score, mean (SD)	0.8 (1.1)	0.8 (1.1)	0.8 (1.0)
Eligible hospital episodes in window, mean (SD)	1.4 (1.2)	1.4 (1.2)	1.4 (0.8)
Observed window duration, days, mean (SD)	68.6 (160.4)	68.6 (160.5)	62.6 (148.2)
Hospital palliative-care coding	46,487 (17.0%)	46,061 (17.0%)	426 (21.4%)
ICU admission	25,474 (9.3%)	25,298 (9.3%)	176 (8.8%)
In-hospital chemotherapy coding	15,777 (5.8%)	15,682 (5.8%)	95 (4.8%)
Mechanical ventilation	7100 (2.6%)	7012 (2.6%)	88 (4.4%)
Transfusion	29,066 (10.7%)	28,856 (10.7%)	210 (10.6%)
Tracheostomy	3243 (1.2%)	3228 (1.2%)	15 (0.8%)

### Primary outcome

Hospital palliative-care coding was recorded during the metastatic care window in 46,487 patients overall, including 426 patients with psychotic-disorder coding. After adjustment, psychotic-disorder coding was associated with higher odds of hospital palliative-care coding during the metastatic care window (OR 1.35, 95% CI 1.19–1.52, *p* < 0.001). The marginal adjusted probability of hospital palliative-care coding was 20.6% with psychotic-disorder coding and 17.0% without psychotic-disorder coding, corresponding to an adjusted risk difference of 3.6 percentage points (Table 4).

**Table 4:** Adjusted association between psychotic-disorder coding and hospital outcomes during the metastatic care window

Outcome	Events with psychotic disorder	Adjusted OR (95% CI)	*p* value	Adj. prob., no psychotic disorder	Adj. prob., psychotic disorder	Adj. risk difference
Hospital palliative-care coding	426 (21.4%)	1.35 (1.19–1.52)	< 0.001	17.0%	20.6%	+ 3.6 pp
ICU admission	176 (8.8%)	1.07 (0.90–1.28)	0.440	9.3%	9.8%	+ 0.5 pp
In-hospital chemotherapy coding	95 (4.8%)	0.73 (0.59–0.91)	0.005	5.8%	4.5%	− 1.3 pp
Mechanical ventilation	88 (4.4%)	1.44 (1.15–1.82)	0.002	2.6%	3.6%	+ 1.0 pp
Transfusion	210 (10.6%)	1.02 (0.88–1.19)	0.755	10.7%	10.9%	+ 0.2 pp
Tracheostomy	15 (0.8%)	0.60 (0.32–1.15)	0.127	1.2%	0.9%	− 0.3 pp

### Secondary outcomes

Psychotic-disorder coding was associated with higher adjusted odds of mechanical ventilation during the metastatic care window (OR 1.44, 95% CI 1.15–1.82, *p* = 0.002). Intensive care unit admission showed no clear association (OR 1.07, 95% CI 0.90–1.28, *p* = 0.440).

In-hospital chemotherapy procedure coding was less frequent among patients with psychotic-disorder coding. After adjustment, psychotic-disorder coding was associated with lower odds of in-hospital chemotherapy procedure coding during the metastatic care window (OR 0.73, 95% CI 0.59–0.91, *p* = 0.005). This outcome should not be interpreted as complete ascertainment of systemic anticancer therapy.

No clear association was observed for transfusion (OR 1.02, 95% CI 0.88–1.19) or tracheostomy (OR 0.60, 95% CI 0.32–1.15) in the primary model.

### Sensitivity analyses

Sensitivity analyses supported the main pattern and are reported in the supplementary material. In the C78-C79 restricted cohort, 205,136 patients were included and 1587 had psychotic-disorder coding; hospital palliative-care coding (OR 1.26, 95% CI 1.11–1.43), in-hospital chemotherapy procedure coding (OR 0.72, 95% CI 0.58–0.91), and mechanical ventilation (OR 1.50, 95% CI 1.17–1.93) remained associated in the same directions. In the sensitivity model defining exposure as F20-F29 coding before or at the first metastatic-coded episode, the corresponding estimates were OR 1.28 (95% CI 1.13–1.45), OR 0.67 (95% CI 0.53–0.84), and OR 1.40 (95% CI 1.10–1.77). In a fixed 12-month sensitivity analysis restricted to patients whose first metastatic-coded episode occurred by 31 December 2023, the corresponding estimates were OR 1.36 (95% CI 1.18–1.57), OR 0.71 (95% CI 0.55–0.91), and OR 1.48 (95% CI 1.13–1.94). Results were also similar in models omitting hospital service and in diagnostic sensitivity analyses focused on schizophrenia/schizoaffective coding (F20/F25) or excluding acute/transient psychosis coding (F23). Tracheostomy was specification-sensitive and was not treated as a central finding.

As an additional audit addressing source-extract consistency, excluding patients whose administrative index episode had Z51.5 as the principal diagnosis did not materially change the main estimates for hospital palliative-care coding (OR 1.34, 95% CI 1.19–1.51), in-hospital chemotherapy procedure coding (OR 0.71, 95% CI 0.57–0.89), or mechanical ventilation (OR 1.45, 95% CI 1.15–1.83).

## Discussion

In this nationwide hospital administrative oncology extract, psychotic-disorder coding was associated with different hospital care patterns during the metastatic care window. Patients with psychotic-disorder coding had higher adjusted odds of hospital palliative-care coding and mechanical ventilation, and lower adjusted odds of in-hospital chemotherapy procedure coding.

The higher likelihood of hospital palliative-care coding may have several interpretations. It may reflect greater symptom burden, higher psychosocial complexity, more frequent recognition of supportive care needs, later presentation with greater clinical deterioration, or different documentation practices during admissions involving psychiatric comorbidity. Because administrative data capture coding rather than direct care quality, this finding should not be interpreted as definitive evidence of better, earlier, more specialist, or more appropriate palliative care access. However, it indicates that psychotic-disorder coding is associated with measurable differences in hospital pathways among patients with metastatic/secondary malignant neoplasm coding.

Our hospital palliative-care coding finding is supported by two particularly relevant French studies in patients with schizophrenia and cancer. Gravier et al. reported higher access to palliative care among cancer patients with schizophrenia in a national multicenter retrospective cohort. Viprey et al., in a French national population-based study of patients with lung cancer and schizophrenia, also found higher use of palliative care among patients with schizophrenia [23, 24]. These studies are aligned with our main finding, while differences in outcome definition and care setting should be kept in mind.

At the same time, the literature is not uniform. Other studies have reported lower access to specialist palliative care, disparities in end-of-life care, or potentially worse care trajectories among patients with psychosis or severe mental illness [25–28]. Taken together, the evidence suggests that the relationship between psychotic disorders and palliative oncology care is not unidirectional. It likely depends on the health system, the setting studied, the type of palliative care measured, and whether the outcome captures specialist palliative care, hospital coding, community care, or end-of-life intensity.

The mechanical ventilation result is notable because it points toward higher coding of an acute life-sustaining intervention. This may reflect acute clinical complexity, delayed presentation, differences in goals-of-care documentation, or greater difficulty aligning care plans before hospital deterioration. The absence of a statistically clear association with ICU admission suggests that ventilation and ICU use may capture overlapping but not identical care processes in administrative data.

The in-hospital chemotherapy procedure coding finding is clinically important but must be interpreted cautiously. The adjusted estimate suggests lower odds of hospital-coded chemotherapy procedures among patients with psychotic-disorder coding. This result is not definitive evidence of undertreatment and does not capture outpatient or day-hospital systemic anticancer therapy. Potential explanations include performance status, comorbidity, contraindications, patient preferences, adherence challenges, fragmented oncology follow-up, poorer care coordination, diagnostic overshadowing, reduced oncology engagement, stigma, or barriers in shared decision-making. Future studies linking administrative records with clinical staging, performance status, treatment intent, psychiatric history, and outpatient oncology data are needed.

Lower in-hospital chemotherapy procedure coding and higher mechanical ventilation are separate group-level associations measured during the metastatic care window. They do not establish that these outcomes occurred in the same individuals, occurred in a specific temporal order, or that one type of care replaced another. The pattern is therefore best interpreted as hypothesis-generating evidence of different hospital coding and care patterns among patients with psychotic-disorder coding.

The C78-C79 sensitivity analysis reduced concern that the findings were driven solely by inclusion of C77 lymph-node secondary malignant neoplasm coding. Similar numbers of eligible hospital episodes and observed window duration by psychotic-disorder coding status also reduce, but do not eliminate, concern that higher palliative-care coding simply reflected more opportunities for coding.

This study has several strengths. It uses a large nationwide administrative dataset, applies the patient as the unit of analysis, focuses on metastatic/secondary malignant neoplasm coding in an oncology hospital extract, and evaluates both hospital palliative-care coding and selected markers of hospital treatment intensity. The analytic choices were defined to be clinically interpretable, particularly the use of a metastatic care window beginning at the first hospital episode with documented secondary malignant neoplasm/metastatic coding.

The study also has limitations. Administrative data are subject to coding variability and cannot confirm treatment intent, appropriateness, symptom burden, performance status, patient preferences, outpatient treatment, care quality, referral timing, or the content of palliative-care interventions. The source extract was an oncology administrative extract composed overwhelmingly of episodes with cancer as the principal diagnosis; it therefore may omit admissions primarily coded for infection, respiratory failure, psychiatric deterioration, or other non-cancer diagnoses. Hospital palliative-care coding should not be interpreted as direct measurement of specialist access, quality of care, or patient-centeredness. Psychotic disorders may be undercoded, especially if psychiatric diagnoses were not clinically salient during cancer admissions. Although most patients with F20-F29 coding in the metastatic care window had such coding before or at the index episode in the observed extract, administrative data cannot establish clinical onset. Patient linkage relied on the official recoded identifier supplied in the RAE-CMBD extract; the dataset did not include documentation allowing us to independently audit the identifier-generation algorithm or verify linkage stability across hospitals, autonomous communities, and calendar years. The RAE-CMBD extract did not include a hospital identifier or death date, preventing hospital-level cluster-robust inference and formal survival or competing-risk analysis. Chemotherapy administered outside hospital admission may not be captured. Residual confounding is possible.

## Conclusion

Among patients with metastatic/secondary malignant neoplasm coding in a Spanish hospital oncology administrative extract, psychotic-disorder coding was associated with higher hospital palliative-care coding and mechanical ventilation, and with lower in-hospital chemotherapy procedure coding. These findings identify distinct hospital coding patterns and support further study of integrated oncology, psychiatry, and palliative-care pathways for patients with advanced cancer and psychotic disorders.

## Supplementary Information

Below is the link to the electronic supplementary material.

ESM 1 DOCX (37.4 KB)

## Data Availability

The individual-level RAE-CMBD data used in this study are not publicly available because they contain confidential health information and are subject to data-use restrictions. Aggregated outputs may be made available by the corresponding author upon reasonable request, subject to institutional and legal approvals.

## Footnotes

1 The source of the RAE-CMBD data is the Subdirección General de Información Sanitaria of the Spanish Ministry of Health. The accuracy and reliability of the information derived from analyses conducted by the authors are the sole responsibility of the authors.

## Code availability

The analytic code used to generate the study cohort, tables and models is available from the corresponding author upon reasonable request.
