# Supplementary material for: Hospital care patterns among patients with psychotic-disorder coding and metastatic/secondary malignant neoplasm coding: A nationwide administrative cohort study in Spain
Source: Support Care Cancer. 2026 Nov 4;34(11):1193. doi: 10.1007/s00520-026-11440-0 (PMC13736351; doi:10.1007/s00520-026-11440-0)
Supplement: Supplementary file 1 [file 520_2026_11440_MOESM1_ESM.docx]

# Supplementary Material

Hospital care patterns among patients with psychotic-disorder coding and metastatic/secondary malignant neoplasm coding: a nationwide administrative cohort study in Spain

Belén Sánchez-Barriguete

Carlos Navarro-Garcí­a

# Supplementary Methods

## Source Extract

The source dataset was a Spanish RAE-CMBD administrative hospital episode extract covering 2021-2024. The extract was requested as an oncology dataset and was composed overwhelmingly of hospital episodes with malignant cancer recorded as the principal diagnosis: 1,206,045 of 1,224,317 episodes (98.5%). A small subset of episodes in the supplied extract had palliative-care coding (Z51.5/Z515) as the principal diagnosis with malignant neoplasm/metastatic coding in secondary fields. It should therefore not be interpreted as all patients with metastatic cancer in Spain or all hospitalizations experienced by patients with metastatic cancer.

The patient identifier used for linkage was the recoded patient identifier supplied in the RAE-CMBD extract. The analysis unit was the patient. We treated this official linkage variable as the longitudinal patient key across observed episodes; the extract did not include documentation allowing us to independently audit the identifier-generation algorithm or verify linkage stability across hospitals, autonomous communities, and calendar years. The extract did not contain a hospital identifier or a validated death date. Autonomous community and discharge-status fields were available.

## Cohort Definition

The primary cohort included patients with at least one secondary diagnosis code beginning with C77, C78, or C79 in secondary diagnosis fields 2 to 20.

The first observed hospital episode with one of these secondary malignant neoplasm/metastatic disease codes was treated as the administrative index episode. This index episode does not necessarily represent the onset of metastatic disease. Patients may have had newly documented or prevalent metastatic disease.

In the final analytic cohort, 4,814 patients (1.8%) had Z51.5/Z515 as the principal diagnosis at the administrative index episode and C77-C79 metastatic/secondary malignant neoplasm coding in secondary diagnosis fields. These patients were retained in the primary analysis because they met the metastatic/secondary malignant neoplasm coding criterion and represented hospital palliative-care principal-diagnosis episodes within the supplied oncology extract.

Because C77 may include lymph-node secondary malignant neoplasm coding that does not always represent distant stage IV disease, we performed a sensitivity analysis restricted to patients with C78-C79 coding.

Supplementary Table S1. Overlap between C77 and C78-C79 coding during the metastatic care window.

| Group | Patients |
| --- | --- |
| Any C77 in window, no C78-C79 in window | 67,673 |
| Any C78-C79 in window | 205,136 |
| Both C77 and C78-C79 in window | 64,025 |
| Index episode C77 only | 73,095 |
| Index episode C78-C79 | 199,714 |

## Observation Window

For each patient, the metastatic care window started at the first observed metastatic/secondary malignant neoplasm-coded hospital episode and ended at the last available observed hospital episode for that patient within 2021-2024.

Outcomes were considered present if coded at least once within this window. Observation duration was calculated from first metastatic-coded admission date to the last contact date where available; otherwise the last admission date was used.

## Exposure Definition

The primary exposure was psychotic-disorder coding, defined as any secondary diagnosis code beginning with F20-F29 within the metastatic care window. A temporal audit also identified F20-F29 coding before or at the first metastatic-coded episode in the observed extract.

The F20-F29 group includes schizophrenia, schizotypal disorder, persistent delusional disorders, acute and transient psychotic disorders, schizoaffective disorders, and other non-organic psychotic disorders. Sensitivity analyses were specified for schizophrenia/schizoaffective coding (F20 or F25) and for excluding acute/transient psychosis (F23).

Supplementary Table S2. Distribution of psychotic-disorder diagnostic code groups among patients with F20-F29 coding in the metastatic care window.

| Code group | Patients |
| --- | --- |
| F20 | 1,421 |
| F21 | 14 |
| F22 | 221 |
| F23 | 18 |
| F24 | 0 |
| F25 | 184 |
| F26 | 0 |
| F27 | 0 |
| F28 | 2 |
| F29 | 164 |

*Note:* Diagnostic code groups are not mutually exclusive; therefore, counts may exceed the number of patients with any F20-F29 coding.

## Outcome Definitions

Hospital palliative-care coding was defined by diagnosis code Z51.5, stored without punctuation as Z515, in any diagnosis field. This measure indicates palliative-care coding within a hospital episode. It does not identify specialist palliative-care referral, timing, content, intensity, quality, or preference-concordance.

Intensive care unit admission was defined by the administrative ICU admission field equal to 1.

In-hospital chemotherapy procedure coding was defined using ICD-10-PCS-derived procedure pattern 3E0..0. after punctuation removal. This does not capture outpatient chemotherapy, day-hospital treatment, immunotherapy, targeted therapy, oral anticancer therapy, or all systemic anticancer treatment.

Mechanical ventilation procedure coding was defined as any ICD-10-PCS-derived procedure code beginning with 5A0 or 5A19.

Transfusion procedure coding was defined as any ICD-10-PCS-derived procedure code beginning with 3023.

Tracheostomy procedure coding was defined as any ICD-10-PCS-derived procedure code beginning with 0B110, 0B113, 0B114, or 0B11F.

## Covariates

Age, sex, calendar year, autonomous community, hospital service, primary tumor group, and approximate non-oncologic Charlson-based comorbidity score were measured at the first metastatic-coded episode.

Primary tumor groups were derived from the principal diagnosis at the first metastatic-coded episode and grouped as colorectal, lung, breast, pancreas, stomach, prostate, liver, hematologic, esophagus, larynx, melanoma, ovary, kidney, central nervous system/brain, thyroid, uterus/cervix, bladder, other cancer, or other.

The comorbidity measure used in the models was an approximate non-oncologic Charlson-based ICD-10 prefix algorithm using secondary diagnosis fields and excluding malignancy/metastasis components. This measure was not a fully validated Quan/Charlson implementation and should be interpreted as an administrative comorbidity approximation.

## Operational Definitions

The following table summarizes the source fields and operational rules used for the main cohort, exposure, outcomes, tumor groups, hospital-service covariate, and approximate non-oncologic Charlson-based score. Diagnosis and procedure codes were standardized by trimming whitespace, converting to upper case, and removing punctuation before applying prefix or exact-code rules.

Supplementary Table S3. Operational definitions and code rules used in the analysis.

| Variable | Source field | Codes/prefixes/values | Operational rule |
| --- | --- | --- | --- |
| Source extract | Episode inclusion | Requested oncology administrative extract | Hospital episodes in the requested oncology extract for 2021-2024; 98.5% had malignant cancer recorded as the principal diagnosis, and a small subset had palliative-care coding (Z51.5/Z515) as the principal diagnosis with malignant neoplasm/metastatic coding in secondary fields. The extract should not be described as restricted exclusively to malignant-cancer principal diagnoses. |
| Patient identifier | Recoded patient identifier | Recoded patient key | Used as the longitudinal patient identifier across observed hospital episodes. |
| Primary cohort | Secondary diagnosis fields 2-20 | C77, C78, C79 | At least one secondary diagnosis beginning with C77-C79. |
| C78-C79 sensitivity cohort | Secondary diagnosis fields 2-20 | C78, C79 | At least one secondary diagnosis beginning with C78-C79 during the metastatic care window. |
| Administrative index episode | Admission date; secondary diagnosis fields 2-20 | C77, C78, C79 | First observed hospital episode with secondary malignant neoplasm/metastatic coding. |
| Metastatic care window | Admission date; contact end date | Index episode to last observed episode | Patient-level window from first metastatic-coded episode to last available hospital episode/contact date. |
| Psychotic-disorder coding | Secondary diagnosis fields 2-20 | F20-F29 | Any secondary diagnosis beginning with F20-F29 within the metastatic care window. |
| Prior-or-index psychotic-disorder coding | Secondary diagnosis fields 2-20; admission date | F20-F29 | Any secondary diagnosis beginning with F20-F29 before or at the administrative index episode in the observed extract. |
| Schizophrenia/schizoaffective sensitivity | Secondary diagnosis fields 2-20 | F20, F25 | Sensitivity exposure using schizophrenia or schizoaffective disorder coding. |
| Excluding acute/transient psychosis | Secondary diagnosis fields 2-20 | F23 | Sensitivity analysis excluding patients with acute/transient psychosis coding. |
| Hospital palliative-care coding | Any diagnosis field | Z51.5/Z515 | Any palliative-care diagnosis code during the metastatic care window. |
| ICU admission | ICU admission field | 1 | Administrative ICU field equal to 1 during the metastatic care window. |
| In-hospital chemotherapy procedure coding | Procedure fields 1-20 | 3E0..0. after punctuation removal | Any procedure code matching the prespecified in-hospital chemotherapy procedure pattern. |
| Mechanical ventilation | Procedure fields 1-20 | 5A0, 5A19 | Any procedure code beginning with 5A0 or 5A19 during the metastatic care window. |
| Transfusion | Procedure fields 1-20 | 3023 | Any procedure code beginning with 3023 during the metastatic care window. |
| Tracheostomy | Procedure fields 1-20 | 0B110, 0B113, 0B114, 0B11F | Any procedure code beginning with one of the listed prefixes during the metastatic care window. |
| Hospital service | Hospital service field | Original service code | Service recorded at the first metastatic-coded episode; used as categorical covariate without further grouping, with missing values retained as Missing. |
| Autonomous community | Autonomous community field | Original territorial code | Autonomous community recorded at the first metastatic-coded episode; included as categorical covariate. |
| Sex | Sex field | 1 male; 2 female; 9/not available not recorded | Sex recorded at the first metastatic-coded episode; not-recorded values retained explicitly. |
| Index year | Year field | 2021, 2022, 2023, 2024 | Calendar year of the first metastatic-coded episode. |
| Tumor group: colorectal | Principal diagnosis field | C18, C19, C20 | Principal diagnosis at index episode begins with C18-C20. |
| Tumor group: lung | Principal diagnosis field | C33, C34 | Principal diagnosis at index episode begins with C33-C34. |
| Tumor group: breast | Principal diagnosis field | C50 | Principal diagnosis at index episode begins with C50. |
| Tumor group: pancreas | Principal diagnosis field | C25 | Principal diagnosis at index episode begins with C25. |
| Tumor group: stomach | Principal diagnosis field | C16 | Principal diagnosis at index episode begins with C16. |
| Tumor group: prostate | Principal diagnosis field | C61 | Principal diagnosis at index episode begins with C61. |
| Tumor group: liver | Principal diagnosis field | C22 | Principal diagnosis at index episode begins with C22. |
| Tumor group: hematologic | Principal diagnosis field | C81-C86, C88, C90-C96 | Principal diagnosis at index episode begins with one of the listed hematologic malignancy prefixes. |
| Tumor group: esophagus | Principal diagnosis field | C15 | Principal diagnosis at index episode begins with C15. |
| Tumor group: larynx | Principal diagnosis field | C32 | Principal diagnosis at index episode begins with C32. |
| Tumor group: melanoma | Principal diagnosis field | C43 | Principal diagnosis at index episode begins with C43. |
| Tumor group: ovary | Principal diagnosis field | C56 | Principal diagnosis at index episode begins with C56. |
| Tumor group: kidney | Principal diagnosis field | C64 | Principal diagnosis at index episode begins with C64. |
| Tumor group: CNS/brain | Principal diagnosis field | C70, C71, C72 | Principal diagnosis at index episode begins with C70-C72. |
| Tumor group: thyroid | Principal diagnosis field | C73 | Principal diagnosis at index episode begins with C73. |
| Tumor group: uterus/cervix | Principal diagnosis field | C53, C54, C55 | Principal diagnosis at index episode begins with C53-C55. |
| Tumor group: bladder | Principal diagnosis field | C67 | Principal diagnosis at index episode begins with C67. |
| Tumor group: other cancer | Principal diagnosis field | Other C codes | Principal diagnosis starts with C and is not assigned to another listed tumor group. |
| Tumor group: other | Principal diagnosis field | Non-C/other | Principal diagnosis not assigned to a malignant cancer group. In the final analytic cohort, this category comprised Z51.5/Z515 palliative-care principal-diagnosis episodes with C77-C79 metastatic/secondary malignant neoplasm coding in secondary diagnosis fields. |
| Non-oncologic Charlson: Myocardial infarction | Secondary diagnosis fields 2-20 at index episode | I21, I22, I25 | Weight 1 |
| Non-oncologic Charlson: Congestive heart failure | Secondary diagnosis fields 2-20 at index episode | I50 | Weight 1 |
| Non-oncologic Charlson: Peripheral vascular disease | Secondary diagnosis fields 2-20 at index episode | I70, I71, I73 | Weight 1 |
| Non-oncologic Charlson: Cerebrovascular disease | Secondary diagnosis fields 2-20 at index episode | I60-I64, I69 | Weight 1 |
| Non-oncologic Charlson: Dementia | Secondary diagnosis fields 2-20 at index episode | F00-F03, G30 | Weight 1 |
| Non-oncologic Charlson: Chronic pulmonary disease | Secondary diagnosis fields 2-20 at index episode | J40-J47 | Weight 1 |
| Non-oncologic Charlson: Rheumatic disease | Secondary diagnosis fields 2-20 at index episode | M05, M06, M32-M34 | Weight 1 |
| Non-oncologic Charlson: Peptic ulcer disease | Secondary diagnosis fields 2-20 at index episode | K25-K28 | Weight 1 |
| Non-oncologic Charlson: Mild liver disease | Secondary diagnosis fields 2-20 at index episode | K70, K71, K73, K74, K76 | Weight 1 |
| Non-oncologic Charlson: Diabetes | Secondary diagnosis fields 2-20 at index episode | E10-E14 | Weight 1 |
| Non-oncologic Charlson: Hemiplegia/paraplegia | Secondary diagnosis fields 2-20 at index episode | G81, G82 | Weight 2 |
| Non-oncologic Charlson: Renal disease | Secondary diagnosis fields 2-20 at index episode | N18, N19 | Weight 2 |
| Non-oncologic Charlson: Moderate/severe liver disease | Secondary diagnosis fields 2-20 at index episode | K72 | Weight 3 |
| Non-oncologic Charlson: Excluded Charlson cancer components | Secondary diagnosis fields 2-20 at index episode | C00-C97, metastatic malignancy components | Not included in the approximate non-oncologic Charlson-based score because the cohort was cancer/metastatic-code defined. |

# Supplementary Statistical Analyses

The main model was logistic regression for each binary outcome, with psychotic-disorder coding plus age, sex, index year, tumor group, autonomous community, hospital service, and approximate non-oncologic Charlson-based comorbidity.

Categorical covariates were included using indicator variables. Patients with missing age were excluded from complete-case model fitting; missing or unavailable categorical values were retained as explicit missing/not-recorded categories where applicable.

Marginal adjusted probabilities were estimated by setting psychotic-disorder coding to present and absent for all patients in the fitted model and averaging predicted probabilities. Adjusted risk differences were calculated as the difference between these marginal probabilities.

Hospital-level cluster-robust standard errors could not be estimated because no hospital identifier was available. Models adjusted for autonomous community, but residual hospital-level clustering and coding-practice variation remain possible.

Secondary outcomes were treated as exploratory.

All reported logistic regression models converged. Sparse exploratory outcomes, particularly tracheostomy, were interpreted cautiously because low event counts can make estimates less stable.

# Supplementary Results

## Expanded Patient Characteristics

Supplementary Table S4. Patient characteristics and observation opportunity by psychotic-disorder coding status.

| Characteristic | Total | No psychotic disorder | Psychotic disorder |
| --- | --- | --- | --- |
| Patients, n | 272,809 | 270,820 | 1,989 |
| Age, mean (SD) | 67.3 (13.7) | 67.4 (13.7) | 62.9 (11.2) |
| Sex: Male | 143,810 (52.7%) | 142,672 (52.7%) | 1,138 (57.2%) |
| Sex: Female | 128,997 (47.3%) | 128,146 (47.3%) | 851 (42.8%) |
| Sex: Not recorded | 2 (0.0%) | 2 (0.0%) | 0 (0.0%) |
| Index year: 2021 | 72,234 (26.5%) | 71,716 (26.5%) | 518 (26.0%) |
| Index year: 2022 | 68,024 (24.9%) | 67,549 (24.9%) | 475 (23.9%) |
| Index year: 2023 | 65,960 (24.2%) | 65,477 (24.2%) | 483 (24.3%) |
| Index year: 2024 | 66,591 (24.4%) | 66,078 (24.4%) | 513 (25.8%) |
| Tumor group: Other cancer | 67,987 (24.9%) | 67,564 (24.9%) | 423 (21.3%) |
| Tumor group: Lung | 48,160 (17.7%) | 47,590 (17.6%) | 570 (28.7%) |
| Tumor group: Colorectal | 40,250 (14.8%) | 39,996 (14.8%) | 254 (12.8%) |
| Tumor group: Breast | 28,940 (10.6%) | 28,736 (10.6%) | 204 (10.3%) |
| Tumor group: Pancreas | 17,399 (6.4%) | 17,292 (6.4%) | 107 (5.4%) |
| Tumor group: Stomach | 11,530 (4.2%) | 11,465 (4.2%) | 65 (3.3%) |
| Tumor group: Prostate | 8,529 (3.1%) | 8,502 (3.1%) | 27 (1.4%) |
| Tumor group: Bladder | 7,915 (2.9%) | 7,869 (2.9%) | 46 (2.3%) |
| Tumor group: Ovary | 6,941 (2.5%) | 6,902 (2.5%) | 39 (2.0%) |
| Tumor group: Uterus/cervix | 5,914 (2.2%) | 5,850 (2.2%) | 64 (3.2%) |
| Tumor group: Liver | 5,566 (2.0%) | 5,535 (2.0%) | 31 (1.6%) |
| Tumor group: Other | 4,814 (1.8%) | 4,775 (1.8%) | 39 (2.0%) |
| Tumor group: Thyroid | 4,811 (1.8%) | 4,792 (1.8%) | 19 (1.0%) |
| Tumor group: Kidney | 4,557 (1.7%) | 4,531 (1.7%) | 26 (1.3%) |
| Tumor group: Esophagus | 3,331 (1.2%) | 3,300 (1.2%) | 31 (1.6%) |
| Tumor group: Larynx | 2,064 (0.8%) | 2,045 (0.8%) | 19 (1.0%) |
| Tumor group: Melanoma | 1,884 (0.7%) | 1,876 (0.7%) | 8 (0.4%) |
| Tumor group: Hematologic | 1,468 (0.5%) | 1,461 (0.5%) | 7 (0.4%) |
| Tumor group: CNS/brain | 749 (0.3%) | 739 (0.3%) | 10 (0.5%) |
| Approximate non-oncologic Charlson-based score, mean (SD) | 0.8 (1.1) | 0.8 (1.1) | 0.8 (1.0) |
| Eligible hospital episodes in window, mean (SD) | 1.4 (1.2) | 1.4 (1.2) | 1.4 (0.8) |
| Observed window duration, days, mean (SD) | 68.6 (160.4) | 68.6 (160.5) | 62.6 (148.2) |

**Supplementary Table S5. Distribution of autonomous community and hospital service by psychotic-disorder coding status.**

Autonomous community and hospital service were measured at the first metastatic-coded episode and included as categorical covariates in the primary adjustment model. No blank values were present for either variable. Coded values such as ZZZ are retained as supplied administrative categories and are shown explicitly rather than recoded as blank missing values.

| **Variable** | **Category** | **Total** | **No psychotic-disorder coding** | **Psychotic-disorder coding** |
| --- | --- | --- | --- | --- |
| Autonomous community | 1 - Andalusia | 43,700 (16.0%) | 43,406 (16.0%) | 294 (14.8%) |
| Autonomous community | 2 - Aragon | 9,027 (3.3%) | 8,958 (3.3%) | 69 (3.5%) |
| Autonomous community | 3 - Asturias | 6,591 (2.4%) | 6,546 (2.4%) | 45 (2.3%) |
| Autonomous community | 4 - Balearic Islands | 5,506 (2.0%) | 5,463 (2.0%) | 43 (2.2%) |
| Autonomous community | 5 - Canary Islands | 11,588 (4.2%) | 11,493 (4.2%) | 95 (4.8%) |
| Autonomous community | 6 - Cantabria | 4,096 (1.5%) | 4,079 (1.5%) | 17 (0.9%) |
| Autonomous community | 7 - Castile and Leon | 17,251 (6.3%) | 17,098 (6.3%) | 153 (7.7%) |
| Autonomous community | 8 - Castile-La Mancha | 9,315 (3.4%) | 9,258 (3.4%) | 57 (2.9%) |
| Autonomous community | 9 - Catalonia | 40,991 (15.0%) | 40,612 (15.0%) | 379 (19.1%) |
| Autonomous community | 10 - Valencian Community | 30,275 (11.1%) | 30,091 (11.1%) | 184 (9.3%) |
| Autonomous community | 11 - Extremadura | 5,441 (2.0%) | 5,405 (2.0%) | 36 (1.8%) |
| Autonomous community | 12 - Galicia | 20,412 (7.5%) | 20,268 (7.5%) | 144 (7.2%) |
| Autonomous community | 13 - Madrid | 40,099 (14.7%) | 39,851 (14.7%) | 248 (12.5%) |
| Autonomous community | 14 - Murcia | 7,489 (2.7%) | 7,439 (2.7%) | 50 (2.5%) |
| Autonomous community | 15 - Navarre | 3,813 (1.4%) | 3,781 (1.4%) | 32 (1.6%) |
| Autonomous community | 16 - Basque Country | 14,165 (5.2%) | 14,045 (5.2%) | 120 (6.0%) |
| Autonomous community | 17 - La Rioja | 2,619 (1.0%) | 2,601 (1.0%) | 18 (0.9%) |
| Autonomous community | 18 - Ceuta | 234 (0.1%) | 234 (0.1%) | 0 (0.0%) |
| Autonomous community | 19 - Melilla | 197 (0.1%) | 192 (0.1%) | 5 (0.3%) |
| Hospital service | MIR - Internal medicine | 61,414 (22.5%) | 60,825 (22.5%) | 589 (29.6%) |
| Hospital service | ONC - Medical oncology | 54,742 (20.1%) | 54,404 (20.1%) | 338 (17.0%) |
| Hospital service | CIR - General surgery | 52,767 (19.3%) | 52,492 (19.4%) | 275 (13.8%) |
| Hospital service | OBG - Obstetrics and gynecology | 20,889 (7.7%) | 20,749 (7.7%) | 140 (7.0%) |
| Hospital service | NML - Pulmonology | 12,653 (4.6%) | 12,452 (4.6%) | 201 (10.1%) |
| Hospital service | UCP - Palliative care unit | 12,643 (4.6%) | 12,533 (4.6%) | 110 (5.5%) |
| Hospital service | URO - Urology | 11,904 (4.4%) | 11,854 (4.4%) | 50 (2.5%) |
| Hospital service | DIG - Digestive diseases | 11,870 (4.4%) | 11,779 (4.3%) | 91 (4.6%) |
| Hospital service | CTO - Thoracic surgery | 6,866 (2.5%) | 6,833 (2.5%) | 33 (1.7%) |
| Hospital service | ORL - Otorhinolaryngology | 4,605 (1.7%) | 4,576 (1.7%) | 29 (1.5%) |
| Hospital service | GRT - Geriatrics | 2,614 (1.0%) | 2,597 (1.0%) | 17 (0.9%) |
| Hospital service | CMF - Maxillofacial surgery | 2,411 (0.9%) | 2,406 (0.9%) | 5 (0.3%) |
| Hospital service | CPL - Plastic surgery | 2,221 (0.8%) | 2,215 (0.8%) | 6 (0.3%) |
| Hospital service | URG - Emergency department | 1,729 (0.6%) | 1,713 (0.6%) | 16 (0.8%) |
| Hospital service | ONR - Radiation oncology | 1,532 (0.6%) | 1,528 (0.6%) | 4 (0.2%) |
| Hospital service | NRC - Neurosurgery | 1,296 (0.5%) | 1,287 (0.5%) | 9 (0.5%) |
| Hospital service | HEM - Hematology | 1,237 (0.5%) | 1,230 (0.5%) | 7 (0.4%) |
| Hospital service | MNU - Nuclear medicine | 1,173 (0.4%) | 1,172 (0.4%) | 1 (0.1%) |
| Hospital service | ZZZ - Not specified/unknown service code | 1,164 (0.4%) | 1,157 (0.4%) | 7 (0.4%) |
| Hospital service | MIV - Intensive care medicine | 1,083 (0.4%) | 1,068 (0.4%) | 15 (0.8%) |
| Hospital service | TRA - Traumatology/orthopedics | 767 (0.3%) | 765 (0.3%) | 2 (0.1%) |
| Hospital service | NRL - Neurology | 680 (0.2%) | 675 (0.2%) | 5 (0.3%) |
| Hospital service | ANR - Anesthesiology/resuscitation | 547 (0.2%) | 547 (0.2%) | 0 (0.0%) |
| Hospital service | OTR - Other service | 516 (0.2%) | 509 (0.2%) | 7 (0.4%) |
| Hospital service | RDT - Radiotherapy | 447 (0.2%) | 442 (0.2%) | 5 (0.3%) |
| Hospital service | PED - Pediatrics | 390 (0.1%) | 390 (0.1%) | 0 (0.0%) |
| Hospital service | END - Endocrinology | 373 (0.1%) | 373 (0.1%) | 0 (0.0%) |
| Hospital service | NEF - Nephrology | 308 (0.1%) | 306 (0.1%) | 2 (0.1%) |
| Hospital service | INF - Infectious diseases | 266 (0.1%) | 260 (0.1%) | 6 (0.3%) |
| Hospital service | DER - Dermatology | 251 (0.1%) | 249 (0.1%) | 2 (0.1%) |
| Hospital service | UCE - Short-stay unit | 246 (0.1%) | 245 (0.1%) | 1 (0.1%) |
| Hospital service | UDO - Pain unit | 228 (0.1%) | 225 (0.1%) | 3 (0.2%) |
| Hospital service | CPE - administrative service code as supplied | 215 (0.1%) | 215 (0.1%) | 0 (0.0%) |
| Hospital service | CAR - Cardiology | 185 (0.1%) | 184 (0.1%) | 1 (0.1%) |
| Hospital service | ACV - administrative service code as supplied | 134 (0.0%) | 131 (0.0%) | 3 (0.2%) |
| Hospital service | REU - Rheumatology | 99 (0.0%) | 99 (0.0%) | 0 (0.0%) |
| Hospital service | RAD - Radiology | 73 (0.0%) | 73 (0.0%) | 0 (0.0%) |
| Hospital service | ULE - administrative service code as supplied | 63 (0.0%) | 63 (0.0%) | 0 (0.0%) |
| Hospital service | UTR - administrative service code as supplied | 47 (0.0%) | 47 (0.0%) | 0 (0.0%) |
| Hospital service | OFT - Ophthalmology | 41 (0.0%) | 40 (0.0%) | 1 (0.1%) |
| Hospital service | CCA - administrative service code as supplied | 21 (0.0%) | 21 (0.0%) | 0 (0.0%) |
| Hospital service | PSQ - Psychiatry | 21 (0.0%) | 14 (0.0%) | 7 (0.4%) |
| Hospital service | REH - Rehabilitation | 21 (0.0%) | 21 (0.0%) | 0 (0.0%) |
| Hospital service | MIC - Intensive care medicine | 20 (0.0%) | 19 (0.0%) | 1 (0.1%) |
| Hospital service | MIP - administrative service code as supplied | 11 (0.0%) | 11 (0.0%) | 0 (0.0%) |
| Hospital service | UTX - administrative service code as supplied | 10 (0.0%) | 10 (0.0%) | 0 (0.0%) |
| Hospital service | EST - administrative service code as supplied | 7 (0.0%) | 7 (0.0%) | 0 (0.0%) |
| Hospital service | UCR - administrative service code as supplied | 4 (0.0%) | 4 (0.0%) | 0 (0.0%) |
| Hospital service | MIN - administrative service code as supplied | 2 (0.0%) | 2 (0.0%) | 0 (0.0%) |
| Hospital service | ULM - administrative service code as supplied | 2 (0.0%) | 2 (0.0%) | 0 (0.0%) |
| Hospital service | RAI - administrative service code as supplied | 1 (0.0%) | 1 (0.0%) | 0 (0.0%) |

## Additional Source-Extract Consistency Audit

As an additional audit of source-extract consistency, we repeated the primary adjusted models after excluding the 4,814 patients whose administrative index episode had Z51.5/Z515 as the principal diagnosis. The main adjusted estimates did not materially change: hospital palliative-care coding OR 1.34 (95% CI 1.19-1.51), in-hospital chemotherapy procedure coding OR 0.71 (95% CI 0.57-0.89), and mechanical ventilation OR 1.45 (95% CI 1.15-1.83).

## C78-C79 Restricted Sensitivity Analysis

Supplementary Table S6. Sensitivity analysis restricted to patients with C78-C79 coding.

| Outcome | n | Psychotic-disorder coded patients | Adjusted OR (95% CI) | p value |
| --- | --- | --- | --- | --- |
| Hospital palliative-care coding | 205,136 | 1,587 | 1.26 (1.11-1.43) | <0.001 |
| ICU admission | 205,136 | 1,587 | 1.08 (0.88-1.33) | 0.468 |
| In-hospital chemotherapy coding | 205,136 | 1,587 | 0.72 (0.58-0.91) | 0.005 |
| Mechanical ventilation | 205,136 | 1,587 | 1.50 (1.17-1.93) | 0.001 |
| Transfusion | 205,136 | 1,587 | 1.02 (0.87-1.19) | 0.788 |
| Tracheostomy | 205,136 | 1,587 | 0.45 (0.16-1.26) | 0.127 |

## Prior-or-Index Psychotic-Disorder Coding Sensitivity Analysis

The primary exposure was defined within the metastatic care window. To address temporal alignment, we fitted a sensitivity model defining psychotic-disorder coding as F20-F29 recorded before or at the first metastatic-coded episode in the observed extract.

Supplementary Table S7. Sensitivity analysis defining psychotic-disorder coding before or at the first metastatic-coded episode.

| Outcome | n | Psychotic-disorder coded patients | Events with psychotic disorder | Adjusted OR (95% CI) | p value | Adj. risk difference |
| --- | --- | --- | --- | --- | --- | --- |
| Hospital palliative-care coding | 272,809 | 1,938 | 403 (20.8%) | 1.28 (1.13-1.45) | <0.001 | +2.9 pp |
| ICU admission | 272,809 | 1,938 | 167 (8.6%) | 1.03 (0.86-1.23) | 0.766 | +0.2 pp |
| In-hospital chemotherapy coding | 272,809 | 1,938 | 88 (4.5%) | 0.67 (0.53-0.84) | <0.001 | -1.7 pp |
| Mechanical ventilation | 272,809 | 1,938 | 83 (4.3%) | 1.40 (1.10-1.77) | 0.006 | +0.9 pp |
| Transfusion | 272,809 | 1,938 | 196 (10.1%) | 0.98 (0.84-1.14) | 0.756 | -0.2 pp |
| Tracheostomy | 272,809 | 1,938 | 14 (0.7%) | 0.62 (0.32-1.19) | 0.149 | -0.3 pp |

## Fixed 12-Month Sensitivity Analysis

To address unequal opportunities for coding by calendar year of cohort entry, we repeated the models using a fixed 12-month window and restricted the analysis to patients whose index admission date was on or before 31 December 2023. This date-based selection yielded 207,667 patients; it differs from the 206,218 patients obtained by summing the 2021-2023 administrative-year categories because eligibility was determined from the index admission date rather than the administrative year. In this sensitivity analysis, the 365-day window began on and included the index episode (day 0), and both psychotic-disorder exposure (F20-F29 coding) and outcomes were ascertained during the 365-day period comprising the index episode (day 0) and the following 364 days.

Supplementary Table S8. Sensitivity analysis using a fixed 12-month window from and including the first metastatic-coded episode.

| Outcome | n | Psychotic-disorder coded patients | Adjusted OR (95% CI) | p value | Adjusted risk difference |
| --- | --- | --- | --- | --- | --- |
| Hospital palliative-care coding | 207,667 | 1,481 | 1.36 (1.18-1.57) | <0.001 | +3.6 pp |
| ICU admission | 207,667 | 1,481 | 1.07 (0.87-1.32) | 0.519 | +0.5 pp |
| In-hospital chemotherapy coding | 207,667 | 1,481 | 0.71 (0.55-0.91) | 0.007 | -1.4 pp |
| Mechanical ventilation | 207,667 | 1,481 | 1.48 (1.13-1.94) | 0.005 | +1.0 pp |
| Transfusion | 207,667 | 1,481 | 0.98 (0.82-1.16) | 0.812 | -0.2 pp |
| Tracheostomy | 207,667 | 1,481 | 0.62 (0.30-1.29) | 0.204 | -0.3 pp |

## Model Omitting Hospital Service

Hospital service at the first metastatic-coded episode may partly represent the pathway under study. We therefore fitted a sensitivity model omitting hospital service from the adjustment set.

Supplementary Table S9. Sensitivity analysis omitting hospital service from the adjustment set.

| Outcome | Adjusted OR without service (95% CI) | p value |
| --- | --- | --- |
| Hospital palliative-care coding | 1.47 (1.31-1.65) | <0.001 |
| ICU admission | 0.87 (0.74-1.02) | 0.094 |
| In-hospital chemotherapy coding | 0.63 (0.51-0.79) | <0.001 |
| Mechanical ventilation | 1.45 (1.16-1.80) | <0.001 |
| Transfusion | 1.03 (0.89-1.19) | 0.703 |
| Tracheostomy | 0.49 (0.27-0.88) | 0.017 |

## Diagnostic Sensitivity Analyses

Given the diagnostic heterogeneity of F20-F29, additional sensitivity analyses were conducted focusing on schizophrenia/schizoaffective coding (F20/F25) and excluding acute/transient psychosis coding (F23). These models omit hospital service and should be interpreted as sensitivity analyses, not as replacements for the primary model.

Supplementary Table S10. Diagnostic sensitivity analyses for heterogeneity within F20-F29 coding.

| Sensitivity analysis | Outcome | Psychotic-disorder coded patients | Adjusted OR (95% CI) | p value |
| --- | --- | --- | --- | --- |
| Schizophrenia/schizoaffective coding (F20/F25) | Hospital palliative-care coding | 1,596 | 1.47 (1.29-1.68) | <0.001 |
| Schizophrenia/schizoaffective coding (F20/F25) | ICU admission | 1,596 | 0.89 (0.75-1.07) | 0.209 |
| Schizophrenia/schizoaffective coding (F20/F25) | In-hospital chemotherapy coding | 1,596 | 0.64 (0.50-0.80) | <0.001 |
| Schizophrenia/schizoaffective coding (F20/F25) | Mechanical ventilation | 1,596 | 1.53 (1.21-1.93) | <0.001 |
| Schizophrenia/schizoaffective coding (F20/F25) | Transfusion | 1,596 | 0.97 (0.82-1.14) | 0.683 |
| Schizophrenia/schizoaffective coding (F20/F25) | Tracheostomy | 1,596 | 0.60 (0.33-1.10) | 0.099 |
| Excluding acute/transient psychosis (F23) | Hospital palliative-care coding | 1,971 | 1.49 (1.33-1.67) | <0.001 |
| Excluding acute/transient psychosis (F23) | ICU admission | 1,971 | 0.87 (0.74-1.02) | 0.083 |
| Excluding acute/transient psychosis (F23) | In-hospital chemotherapy coding | 1,971 | 0.64 (0.51-0.79) | <0.001 |
| Excluding acute/transient psychosis (F23) | Mechanical ventilation | 1,971 | 1.48 (1.20-1.84) | <0.001 |
| Excluding acute/transient psychosis (F23) | Transfusion | 1,971 | 1.02 (0.88-1.18) | 0.774 |
| Excluding acute/transient psychosis (F23) | Tracheostomy | 1,971 | 0.47 (0.26-0.86) | 0.014 |

## Temporal Audit of Psychotic-Disorder Coding

In the primary analytic cohort, psychotic-disorder coding was present in 1,989 of 272,809 patients (0.73%) when defined within the metastatic care window. A temporal audit identified 1,938 patients with F20-F29 coding before or at the first metastatic-coded episode in the observed extract. These definitions were not fully nested: 1,889 patients had F20-F29 coding both within the metastatic care window and before or at the index episode, 100 had F20-F29 coding in the metastatic care window only after the index episode, and 49 had F20-F29 coding before or at the index episode but not within the metastatic care window.

This audit supports the robustness of the exposure definition within the observed administrative data, but it does not establish the clinical onset date of psychotic disorder.
